# Prevention, Reduction and Repair of Brain Injury of the Preterm Infant

**DOI:** 10.3389/fphys.2019.00181

**Published:** 2019-03-20

**Authors:** Frank van Bel, Josine Vaes, Floris Groenendaal

**Affiliations:** ^1^ Department of Neonatology, Wilhelmina Children’s Hospital and Brain Center Rudolf Magnus, University Medical Center Utrecht, Utrecht University, Utrecht, Netherlands; ^2^ Laboratory of Neuroimmunology and Developmental Origins of Disease, University Medical Center Utrecht, Utrecht University, Utrecht, Netherlands

**Keywords:** prematurity, brain hemorrhage, white matter injury in the preterm infant, neuroprotection, neuroregeneration

## Abstract

Periventricular-intraventricular hemorrhages (PIVH) and (diffuse) white matter injury (WMI) are the most important acquired brain lesions of the very and extremely prematurely born neonate. Both carry a high risk for death or adverse neurodevelopmental outcome. The first part of the review discusses the standard of care and latest insights with respect to prevention and/or reduction of PIVH and WMI, taking into account their etiopathogenesis which is tightly linked to (functional) immaturity of the cerebral vascular bed and nervous system and commonly encountered inflammation. The second part discusses repair of hemorrhagic- ischemic and post-inflammatory brain lesions as it is an increasingly important topic in newborn medicine. In the near future trials of trophic and (autologous or allogenic) cell-therapy in infants at risk of or demonstrating established PIVH and WMI will be started. The focus of these potential trials will be discussed.

## Introduction

**Figure 1:**
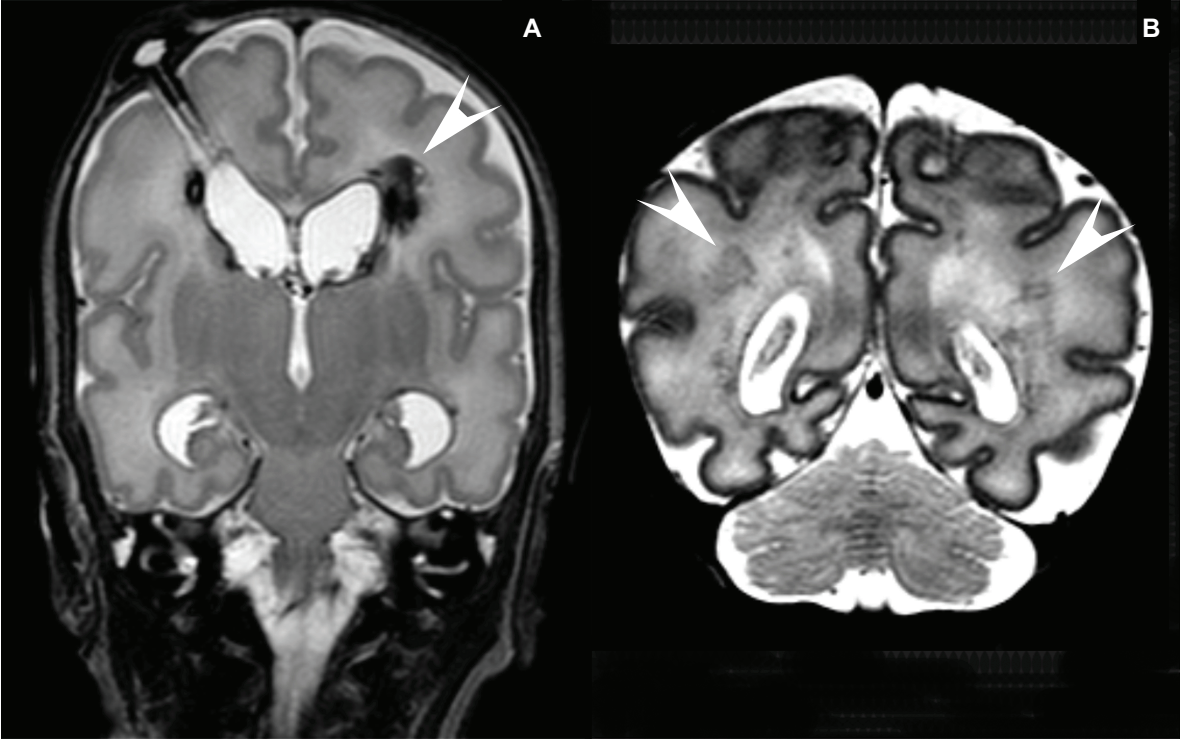
**(A)** MRI of a preterm infant (gestational age 26 2/7 weeks, birthweight 965 g) with a large IVH and a large left-sided (arrowhead) and small right-sided venous infarct. Post-hemorrhagic ventricular dilatation was treated with CSF removal from a subcutaneous reservoir. T2 weighted coronal image at the age of 8 weeks after birth. **(B)** MRI of a preterm infant (gestational age 32 2/7 weeks, birthweight 1,670 g) with several lesions in the periventricular white matter (arrowheads). T2 weighted coronal image at the age of 6 days after birth.

The most important acquired brain injuries in very and extremely preterm infants born in developed countries are periventricular-intraventricular hemorrhages (PIVH) and diffuse white matter injury (dWMI, Figure 1; Stoll et al., 2010; Hamilton et al., 2013; Pierrat et al., 2017). This brain injury may lead to cerebral palsy and learning difficulties, and can have major impact on the quality of life (Stoll et al., 2010; Pierrat et al., 2017).

The first aim of this review is to link the etiopathogenesis of PIVH and dWMI to the standard of care and its latest insights with respect to prevention and reduction of these complications.

The second aim is to focus on repair of the sequelae of PIVH and dWMI. There is increasing evidence that repair of perinatal brain injury with trophic and/or stem cell therapy is currently becoming a realistic and exciting option (Fleiss et al., 2014; Fischer et al., 2017; Wagenaar et al., 2017). We discuss this development in relation with repair of the sequelae of severe PIVH and dWMI.

## Periventricular-Intraventricular Hemorrhage

PIVH has still a high incidence in the developed world: 25–35% of preterm infants born before 30 weeks of gestation or a birth weight less than 1,500 g develop PIVH. PIVH develops from the fragile vascular network of the germinal matrix mostly within the first 3 days after birth with the highest incidence in extremely low birth weight infants being up to 45% (Jain et al., 2009; Stoll et al., 2010; Mukerji et al., 2015). Although a minority of these infants develop severe PIVH grade III (intraventricular blood in dilated lateral cerebral ventricles) or IV (intraventricular blood with extension into the adjacent parenchymal region, more recently described as venous infarction) according to the Papile grading (Papile et al., 1978), up to 75% develop mild to severe PIVH-related sequelae in later life (Sherlock et al., 2005; Luu et al., 2009). PIVH remains therefore a major health concern.

Although multifactorial, the pathogenesis of PIVH and its extension to more severe stages is firmly linked to pulmonary immaturity. This is clinically represented by the idiopathic respiratory distress syndrome (IRDS), and (functional) immaturity of the cerebral vascular bed (Ozdemir et al., 1997; Krediet et al., 2006; Ballabh, 2014). IRDS may lead to hypoxia and hypercapnia, lack of cerebral autoregulation and the need for blood pressure support often causing fluctuations and hyperperfusion of the immature brain of the extremely and very preterm infant (Perlman et al., 1985; van Bel et al., 1987), although this mechanism may also be operative in the moderate and late preterm neonate with IRDS (Thygesen et al., 2016). Cerebral hemodynamic instability often leads to PIVH, mostly originating in the germinal matrix, which has a dense but fragile vasculature (Ballabh, 2014). Moreover, IRDS has been associated with inflammatory processes and oxidative stress in the immature lung. Several studies showed elevated pro-inflammatory cytokines, chemokines and indicators of oxidative stress in broncho-alveolar lavage fluid and blood in very preterm neonates with IRDS (Beresford and Shaw, 2002; Gitto et al., 2004). A recent study showed that intra-amniotic inflammation and postnatal IRDS markedly increased the risk for PIVH (Oh et al., 2018). PIVHs, which develop within 12 hours of age, inflammation may play an important role as indicated by the strong association between early PIVH and pro-inflammatory cytokines and oxidative stress (Krediet et al., 2006; Chisholm et al., 2016; Chevallier et al., 2017; Villamor-Martinez et al., 2018). Finally genetic factors can be related to the occurrence of PIVH, but this issue is beyond the scope of this review (Bilguvar et al., 2009; Harteman et al., 2011; Ballabh, 2014).

### Prevention and Reduction of PIVH: Standard of Care

Prevention and reduction of PIVH starts already in the womb: *maternal corticosteroids* during imminent preterm birth have shown to reduce the occurrence of PIVH and is common practice during preterm labor and imminent preterm birth in most high income countries since the late eighties of the last century (Ment et al., 1995; Ballabh, 2014; Roberts et al., 2017). A recent population study (EPICE Cohort) showed even a risk reduction of up to 50% of severe neonatal injury after antenatal corticosteroids administered shortly before birth (Norman et al., 2017). Mostly betamethasone or dexamethasone are used although there is an ongoing debate about their superiority (Brownfoot et al., 2013). Besides the well proven effect of antenatal steroids on lung maturation with a positive effect on respiratory and hemodynamic systems (Roberts et al., 2017), a maturational effect of steroids on the germinal matrix microvasculature has been postulated (Xu et al., 2008). This will establish a decrease in permeability of the cerebral vasculature and stabilization of the endothelial basement membrane (Hedley-Whyte and Hsu, 1986; Tokida et al., 1990).

As antenatally administered corticosteroids induce lung maturation and pulmonary stabilization, *exogenous surfactant application via* the trachea does so postnatally (McPherson and Wambach, 2018). Surfactant may add therefore to a hemodynamic stabilization of the systemic and cerebral circulation leading to less disturbances of cerebral autoregulatory ability of the vascular bed (Lemmers et al., 2006).

Several studies indicated a decrease in the incidence in PIVH after the introduction of surfactant therapy, especially regarding more severe PIVHs (Walti et al., 1995; Greenough and Ahmed, 2013). An older meta-analysis, however, showed no clear benefits of surfactant therapy on the incidence of PIVH, although there was a tendency for a reduction of severe PIVH (Rojas-Reyes et al., 2012). A recent systematic review and meta-analysis investigating the use of early surfactant, defined as surfactant administration within one hour after birth, with noninvasive ventilation and stress reduction found a decrease in severe PIVH with this strategy (Anand et al., 1999; Isayama et al., 2015; Ng et al., 2017).

*Pharmacologic* interventions aiming to prevent or reduce PIVH are numerous. Muscle paralysis was used in order to minimize swings in cerebral perfusion to influence the incidence of PIVH in artificially ventilated preterm infants. PIVH incidence indeed decreased sharply after muscle paralysis (Perlman et al., 1985). More sophisticated ventilation modalities nowadays, including non-invasive ventilation makes muscle paralysis obsolete (McPherson and Inder, 2017). Phenobarbital sedation did not decrease PIVH incidence (Donn et al., 1981; Bedard et al., 1984). Vitamin E, a potent anti-oxidative agent, reduced the incidence of PIVH but routine use was not encouraged because of serious side effects (Brion et al., 2003). Ethamsylate, which has a stabilizing effect on the vascular basement membrane, was widely investigated in the 1980s, but had no positive effect on the PIVH incidence (Benson et al., 1986).

**Figure 2:**
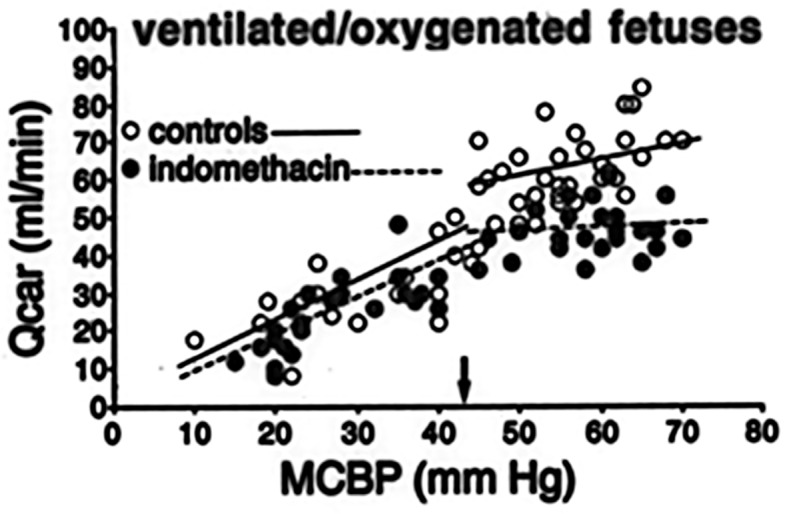
Individual values of Carotid blood flow [Qcar (ml/min)], representing global cerebral blood flow, as a function of (mean) carotid blood pressure (MCBP; mm Hg), representing cerebral perfusion pressure, in pretreated with indomethacin (filled circles) and non-treated ventilated preterm sheep fetuses, representing a perinatal lamb model (van Bel et al., 1993,^1994^,^1995^). Note the lower Qcar values and better autoregulatory curve in the indomethacin-treated animals. The small black arrow indicates the lower limit of MCBP where cerebral autoregulation is still operative.

Only prophylactic indomethacin made its way to the clinic. Indomethacin is a (nonselective) cyclo-oxygenase inhibitor which showed a positive effect on PIVH incidence and induced (early) patent ductus arteriosus closure (Vohr and Ment, 1996). Especially in the United States prophylactic indomethacin administration (low dose indomethacin starting within 6 h after birth up to day 3–5) has been utilized in many centers (Nelin et al., 2017). Although, in 2001 the TIPP trial suggested that despite a decreased incidence of (severe) PIVH, long-term developmental outcome did not improve (Schmidt et al., 2001). A recent large study did show improved survival after indomethacin prophylaxis in especially the extremely preterm infants (Nelin et al., 2017). This seemed to be confirmed by a recent meta-analysis which showed a positive effect on mortality of a prophylactic indomethacin regime (Jensen et al., 2018). It has been suggested that indomethacin promotes maturation of the cerebral vasculature (Ment et al., 1992; Ballabh, 2014). We suggest that also an indomethacin-induced stabilization of cerebral perfusion and improvement of cerebral vascular autoregulation plays a role with respect to reduction of PIVH. Earlier studies of our group in preterm fetal and neonatal lambs showed that indomethacin improved the autoregulatory ability of the cerebrovascular bed, probably due to its vasoconstrictive action, preventing cerebral hyperperfusion as compared to placebo-treated controls (Figure 2; van Bel et al., 1993, 1994, 1995).

*Head position* and especially left or right deviation of the head of very and extremely preterm infants may affect venous drainage by partial occlusion of the jugular vein. This can induce a temporary increase in intracranial pressure. It has been postulated that this may contribute to the occurrence of PIVH (Goldberg et al., 1983).

However, a meta-analysis of relevant studies where the infant was kept supine with the head in the midline position and the bed tilted in 30° to reduce PIVH incidence failed to show a decrease in PIVH incidence as compared to their control counterparts (Romantsik et al., 2017). Additional studies are ongoing.

### Prevention and Reduction of PIVH: Emerging Interventions

Suboptimal blood gas values and hypoxia due to pulmonary immaturity and IRDS play a role in the pathogenesis of PIVH (Ballabh, 2014). Experimental studies and clinical studies using near infrared spectroscopy (NIRS) showed that prolonged episodes of cerebral oxygen saturation lower than 40–45% were related to damage in the developing brain (Dent et al., 2005, Hou et al., 2007). With NIRS-derived monitoring of cerebral oxygenation and perfusion it is possible to timely identify and intervene during episodes of suboptimal oxygenation and perfusion of the immature brain (Skov et al., 1991; van Bel et al., 2008; Wintermark et al., 2014; Alderliesten et al., 2016; van Bel and Mintzer, 2018). Recently, a European randomized controlled multicenter intervention trial (the SafeboosC study) focusing on the reduction of hypoxia and/or hyperoxia, provided evidence that *monitoring cerebral oxygenation* with NIRS lowered the hypoxic burden in extremely preterm neonates in the first days after birth (Hyttel-Sorensen et al., 2015), the episode in which most PIVH occur and/or extend. A follow-up study from this SafeboosC cohort showed that the (early) burden of hypoxia was associated with the occurrence of severe PIVH (Plomgaard et al., 2017). To confirm that interventions on basis of NIRS-monitored cerebral oxygenation can decrease PIVH incidence a contemporary randomized controlled trial with adequate patient inclusions is mandatory. In this respect it is also important to emphasize that clinical application of NIRS in the neonatal intensive care unit, to assess (in) adequacy of cerebral oxygenation, requires international consensus with respect to normative values and understanding of cerebral oxygen utilization patterns (van Bel and Mintzer, 2018).

A potentially promising intervention to lower PIVH incidence is *delayed cord clamping or DCC.* The underlying mechanism may be that a greater neonatal blood volume due to DCC gives rise to an improved cardiac preload leading to a stable cardiac output, stable blood pressure and intact cerebral autoregulation with less need for inotropic therapy (Hooper et al., 2015; Perlman et al., 2015; Wyllie et al., 2015). Consequently the stable hemodynamics may ensure an appropriate cerebral perfusion (Baenziger et al., 2007; Ersdal et al., 2014). Especially lack of cerebral autoregulation and use of positive inotropes seem to be related to a higher incidence and extension of PIVH (Alderliesten et al., 2013). Several studies suggest a positive effect of DCC on PIVH incidence (Rabe et al., 2008, 2012). However, a recent meta-analysis did not yet confirm this although there was a strong tendency for a reducing effect of DCC on PIVH incidence (Fogarty et al., 2018). A key issue with respect to the beneficial effects of DCC on PIVH incidence in very and extremely preterm infants to be solved, is the optimal time of DCC. The delay time in the 27 studies included in the meta-analysis of Fogarty et al was very variable, from 30-up to-more than 120 s (Fogarty et al., 2018). It has been suggested by others that an optimal delay time should be 180 s which may optimize the beneficial effects of DCC (Yao et al., 1969).

Preventive treatment with *trophic factors* and especially *Erythropoietin (EPO)* and *Insulin Growth Factor-1 (IGF-1)* and its *binding protein 3 (IGF-1-BP3)* are increasingly recognized to have neuroprotection and PIVH-reducing properties (Juul and Pet, 2015; Hellstrom et al., 2016).

*EPO* stimulates red cell production, cell survival and differentiation and EPO receptors are detected on endothelial, glial and neuronal cells (van der Kooij et al., 2008; Chateauvieux et al., 2011; Koulnis et al., 2014; Rangarajan and Juul, 2014). EPO has also a modulating effect on glutamate toxicity, stimulating effect on antioxidative ability and anti-inflammatory effect protecting endothelial cells from apoptotic death (Yamaji et al., 1996; Bernaudin et al., 1999; Kawakami et al., 2001). These latter properties of EPO may imply that recombinant human (rh) EPO can also have a positive impact on the PIVH incidence in premature neonates. An older study from Neubauer et al showed indeed a decrease in the incidence of severe PIVH after early rhEPO (Neubauer et al., 2010), although later studies showed conflicting results with respect to PIVH incidence after rhEPO (Ohls et al., 2014; Fauchere et al., 2015). A recent meta-analysis including 3,643 extremely and very preterm infants receiving early EPO therapy reported a reducing effect on PIVH incidence (Fischer et al., 2017; Ohlsson and Aher, 2017).

*IGF-1* is an endogenous protein which exerts several actions: its positive effect on proper vascularization (Hellstrom et al., 2001; Bach, 2015) and brain development are important for a normal neurodevelopment (Hellstrom et al., 2016). Following extremely preterm birth, serum IGF-1 levels are much lower than in utero serum concentrations at corresponding gestational ages. Inadequate endogenous postnatal IGF-1 production is regarded to be the result of preterm birth related events such as hypoxia, inflammation and reduced nutrient availability (Hellstrom et al., 2016). The fact that extremely preterm born infants have deficient serum IGF-1 and IGF-1-BP3 concentrations stimulated researchers and clinicians to perform studies in which suppletion of IGF-1 and its IGF-1 bounding protein BP3 were expected to have maturational effects on vascularization of the extremely preterm neonate (Ley et al., 2013). Intranasal IGF-1 reduced germinal matrix hemorrhages in a preterm rat pup model (Lekic et al., 2016). A clinical study of Hellstrom et al on the effects of IGF-1 on ROP, PIVH and bronchopulmonary dysplasia is ongoing (ClinicalTrials.gov: NCT01096784).

*In summary*, antenatal corticosteroids and the introduction of exogenous surfactant substantially reduced the PIVH incidence of the preterm born infant in high income countries. Better and non-invasive ventilation techniques together with exogenous surfactant treatment and stress reduction during patient care had a further reducing effect on PIVH incidence, as did prophylactic indomethacin treatment.

Promising future therapies for PIVH prevention and/or reduction of severity are delayed cord clamping and early and adequate treatment with trophic factors such as erythropoietin and IGF-1. However, further research is mandatory here. Table 1 shows schematically the above discussed therapeutic considerations.

**Table 1:** Summary of standard care and emerging therapies respectively, for the prevention and reduction of periventricular-intraventricular hemorrhage (PIVH) and (diffuse) white matter injury (dWMI).

PIVH and (d)WMI
Standard care – Antenatal corticosteroids – Exogenous surfactant instillation – Non-invasive ventilation techniques/stress reduction – Prophylactic early (<6 h) indomethacin Emerging therapies – Delayed cord clamping – Trophic factors i.e. erythropoietin (rhEPO) insulin growth factor-1 and its binding protein 3 (IGF-1/IGF-1BP3)

## White Matter Injury in the Very And Extremely Preterm Infant

Extremely preterm born infants (or ELGANs) carry a substantial risk of diffuse white matter injury or abnormal white matter development (Volpe, 2009; Chau et al., 2013). In the early days of neonatal intensive care, white matter injury (WMI; or periventricular leukomalacia) was encountered in the form of cystic periventricular leukomalacia (cPVL), as described by Banker and Larroche (1962). cPVL was hard to detect using CT, but could be detected with the use of cranial ultrasound (cUS), in particular when used longitudinally after the first week after birth (de Vries et al., 2004). The cysts of cPVL appear 10–20 days after an insult, and disappear around term equivalent age. Remaining injury can be seen as widening and irregularity of the ventricles on cUS, and loss of white matter and delayed myelination on cranial MRI (Chau et al., 2013; Martinez-Biarge et al., 2016). Later in life gliosis can be seen in the affected areas. The cysts of cPVL occur alongside the ventricles in preterm infants, whereas subcortical cysts are more common in term infants.

Several causes of cPVL have been suggested, including hypoxia-ischemia and inflammation. Fetal inflammation has been reported to be common in preterm birth (reviewed by Hagberg et al., 2015). Furthermore, preterm CSF appears to show a neuroinflammatory response compared to term infants. Although many have reported white matter injury after maternal chorioamnionitis with infection (reviewed by Paton et al., 2017) (O’Shea et al., 2012; Strunk et al., 2014; Paton et al., 2017), a recent study failed to show a detrimental effect of chorioamnionitis (Bierstone et al., 2018). Reactive oxygen species are considered to play a role in the injury of the cerebral white matter of the preterm infant (Hagberg et al., 2015).

Occurrence of cPVL has been demonstrated after severe hypocapnia and subsequent cerebral vasoconstriction (Groenendaal and de Vries, 2001). The incidence of cPVL is decreasing in modern neonatal intensive care to 1.3% of a NICU cohort of very preterm infants (van Haastert et al., 2011). Probably multiple factors may have contributed to the decrease of cPVL, such as monitoring of blood pressure, low carbon dioxide levels, blood glucose, and cerebral oxygenation using NIRS. The role of maternal antibiotics is still unresolved (Shepherd et al., 2017).

Nowadays, diffuse white matter injury (dWMI), and ‘punctate white matter lesions’ are more commonly seen in extremely preterm infants (Kersbergen et al., 2014a) (Figure 1). Diffuse WMI might even be present in more than 50% of extremely and very preterm infants (Hinojosa-Rodríguez et al., 2017).

A recent review by our group (van Tilborg et al., 2018b), summarizing a substantial amount of preclinical studies, suggested that an arrest in maturation of oligodendrocyte precursors is responsible for hypomyelination as seen in experimental models of dWMI (van de Looij et al., 2012; van Tilborg et al., 2018a). As reviewed by Hagberg et al. (2015) pro-inflammatory cytokines, including IL-6, and TNF-alpha will lead to increased activation of microglia with adverse effects on developing oligodendrocyte precursors. Systemic inflammation in common in extremely and very preterm infants. Although beyond the aim of this review it is important to state that also in moderate and late preterm infants inflammation can lead to brain damage and adverse outcome (Gisslen et al., 2016; Musilova et al., 2018).

Preterm white matter can be studied in far more detail using MRI, and longitudinal scans can visualize brain growth, including growth of specific brain regions, cortical folding and white matter development (Kersbergen et al., 2014b), but identification of tissue microstructure is still challenging (Stolp et al., 2018). Scoring systems have been developed to quantify the abnormalities seen at term equivalent age in this population, and the predictive power for neurodevelopment is under investigation (Inder et al., 2005; Kidokoro et al., 2013). At present, MRI might be more informative in hospitals that are dedicated for neonatal MRI than in general.

### Prevention and Reduction of (Diffuse) White Matter Injury

*Antenatal and perinatal strategies* are very important in the prevention of dWMI. *Magnesium sulphate given antenatally* to women at risk of preterm birth substantially reduced the risk of cerebral palsy of the infant (Crowther et al., 2017). The mechanism of this neuroprotection is still unknown. Improved uterine perfusion through vasodilation, and a reduction of neonatal IVH have been proposed mechanisms. Although magnesium reduces EEG activity and the number of seizures in an animal model of preterm asphyxia (Galinsky et al., 2017; Bennet et al., 2018b), blockade of NMDA receptors or other excitotoxic pathways is unlikely. Although plasma concentrations achieved in mothers and fetuses are increased after maternal administration of magnesium, extracellular magnesium concentrations in the brain are probably lower than those needed for neuroprotection after experimental hypoxia-ischemia.(Crowther et al., 2017; Galinsky et al., 2017).

A recent trial (NCT00724594) tested the pharmacokinetics of maternal and neonatal N-Acetylcysteine. Interestingly, umbilical cord concentrations frequently exceeded maternal concentrations (Wiest et al., 2014). Future studies may aim at the use of N-Acetylcysteine to reduce free radical injury in preterm infants.

*Delayed umbilical cord clamping* has been advised in ‘vigorous’ preterm infants. It is associated with significant neonatal benefits, including improved transitional circulation, better establishment of red blood cell volume, decreased need for blood transfusion, and lower incidence of necrotizing enterocolitis, leading to massive systemic inflammation and subsequent white matter injury, and intraventricular hemorrhage (as already discussed above; (Practice, 2017). Thereby it may have an indirect beneficial effect on white matter injury (see also above: emerging therapies for prevention of PIVH (Mercer et al., 2016)).

*Reduction of severe IRDS* not only reduces IVH (see above), but it may also important in the reduction of severe white matter injury. As through a reduction of severe respiratory illness large fluctuations in oxygen and carbon dioxide levels are avoided, production of reactive oxygen species may be reduced. Furthermore, *stabilization of blood pressure* reduces major swings in cerebral perfusion.

Postnatal pharmacologic interventions for reduction or prevention of dWMI are increasingly recognized as being potentially neuroprotective. Although early *postnatal administration of the corticosteroid dexamethasone* has been reported to be associated with cerebral palsy (Doyle et al., 2017), this may be not the case for *hydrocortisone* (Karemaker et al., 2006). Recently a trial was finished comparing hydrocortisone versus placebo in ventilated preterm infants to reduce chronic lung disease (Onland et al., 2011). Neurodevelopment of these infants will provide information on the benefits (or risks) of postnatal hydrocortisone. Postnatal use of *caffeine* resulted in improved neurodevelopmental outcome (Schmidt et al., 2007). Neonatal caffeine therapy for apnea of prematurity improved visuomotor, visuoperceptual, and visuospatial abilities at age 11 years (Murner-Lavanchy et al., 2018).

It has been suggested that improvement of preterm *nutrition* may contribute to optimizing brain development. In particular the so-called microbiome-gut-brain-*Axis axis* is a proposed mechanism of interaction, including neural, endocrine, and immunological pathways (Cryan and Dinan, 2012). Nutritional components such as fatty acids and protein may stimulate brain growth and neurodevelopment (Uauy and Mena, 2015; Coviello et al., 2018). Also probiotics might be beneficial in reducing the incidence of necrotizing enterocolitis and thereby reduce white matter injury.

*Monitoring of cerebral oxygenation with NIRS* (as already discussed above in relation with prevention of PIVH) and *of brain function* (amplitude EEG [aEEG]), may also play an important preventing role with relation to dWMI.

Since very low arterial CO_2_ levels may contribute to cerebral hypoperfusion and white matter injury (Greisen and Vannucci, 2001). Tools to monitor the neonatal brain oxygenation and function with NIRS and aEEG may contribute to optimize cerebral oxygenation (Hyttel-Sorensen et al., 2015; Plomgaard et al., 2017), and early recognition and treatment of subclinical seizure activity (Glass et al., 2017). Further studies are needed to describe the association with long-term neurodevelopment (Hyttel-Sorensen et al., 2017; Thewissen et al., 2018).

*Pain and stress* are shown to have negative effects on brain development (Duerden et al., 2018). Avoidance of pain appears to be useful. In very preterm infants on mechanical ventilation, continuous fentanyl infusion might protect the developing brain by relieving pain during the first 72 h of mechanical ventilation (Qiu et al., 2018). In contrast others have demonstrated impaired cerebellar growth in the neonatal period and poorer neurodevelopmental outcomes in early childhood of preterm infants after morphine use (Zwicker et al., 2016).

To find an optimal balance between pain and stress reduction and use of opioids may aid in the reduction of white matter injury. Alternative strategies for stress and pain reduction, such as sucrose, use of pacifiers, or non-sedative analgetics need to be explored further.

#### Inflammation

Extremely preterm birth is commonly associated with fetal and postnatal systemic inflammation which is likely to contribute to dWMI through adverse effects on oligodendrocyte precursors (Strunk et al., 2014; Hagberg et al., 2015). Novel strategies are explored to counteract these inflammatory pathways to counteract the deleterious effects on preterm white matter (see below).

### Prevention and Reduction of (d)WMI: Emerging Pharmacologic Interventions

Many anti-inflammatory interventions have been suggested as a result from animal experiments (reviewed by Hagberg et al., 2015). Almost none of these have been tested in human infants.

*Erythropoietin or EPO* has been suggested to inhibit glutamate release, reduce accumulation of intracellular calcium, to induce antiapoptotic factors, to reduce inflammation and nitric oxide-mediated injury, and to contribute to regeneration (van der Kooij et al., 2008; Chateauvieux et al., 2011; Rangarajan and Juul, 2014).

In the EpoKids study in Switzerland very preterm infants were randomized to 3 doses of rhEPO (one before birth, 2 after birth) versus placebo. The secondary outcome of MRI at term equivalent age showed less white matter injury in the EPO group compared with the placebo group (Leuchter et al., 2014). A meta-analysis of administration of rhEPO showed an improved the cognitive development of very preterm infants, as assessed by the MDI at a corrected age of 18–24 months, without affecting other neurodevelopmental outcomes (Fischer et al., 2017). Several trials are still ongoing to study neuroprotection by EPO in preterm infants (Juul and Pet, 2015). Given its positive effect on neurogenesis and angiogenesis a more prolonged course of appropriately (high) dosed rhEPO (up to 2,500 IU/kg daily) may further optimize clinical outcome of the preterm infant (van der Kooij et al., 2008; Chateauvieux et al., 2011; Rangarajan and Juul, 2014).

In animal models *melatonin* has antioxidant properties by influencing several pathways, and reduces (neuro-) inflammation. Through reduction of proinflammatory cytokines pro-oligodendrocyte maturation could be preserved. Administration of *melatonin* to pregnant women with fetal growth restriction or pre-eclampsia is under investigation (NCT02395783 and NCT01695070). Neonatal administration of *melatonin* has been used in preterm newborns with sepsis, surgical procedures or chronic lung disease (Marseglia et al., 2015). However, no beneficial effect on MRI parameters of the preterm brain at term equivalent age could be demonstrated in the relatively low dose administered in this study (Merchant et al., 2014).

*IGF-1* plays a crucial role in fetal and postnatal brain development: IGF-1 is shown to stimulate neurogenesis and proliferation, differentiation and survival of brain cells. Regarding white matter development, IGF-1 also stimulates oligodendrocyte maturation and subsequent myelination (Cao et al., 2003; Pang et al., 2010; Cai et al., 2011; Hansen-Pupp et al., 2011; O’Kusky and Ye, 2012). Moreover, genetic studies in mice display lower total brain volumes and severe hypomyelination following IGF-1 knockout (O’Kusky and Ye, 2012). Human studies relating serum IGF-1 levels to brain development show a positive association between postnatal serum IGF-1 concentrations and head circumference, brain volume measures and developmental scores at 2 years of age (Hansen-Pupp et al., 2011). Main focus of previous studies with IGF-1 and its IGF-1- binding protein 3 was the prevention of retinopathy of prematurity, but the incidence of PIVH will be studied in addition (*ClinicalTrials.gov* *: NCT01096784*). Further studies are needed to explore potential neuroprotective effects of IGF-1 with respect to dWMI.

*In summary,* Injury to and subnormal development of the periventricular white matter is still very common in extremely preterm born infants. Although improved neonatal intensive care may contribute to improved outcomes, additional strategies to counteract (d) WMI may add to an improved neurodevelopmental outcome.

## Repair of Sequelae of Pivh and dWMI

**Figure 3:**
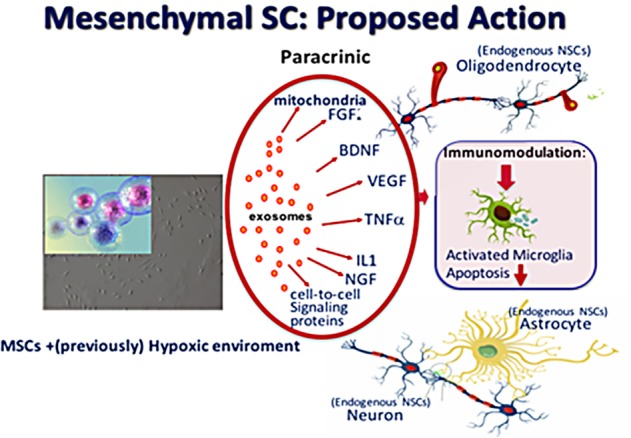
Proposed actions of mesenchymal stem cells (MSC) when present in a (previously) hypoxic–ischemic environment: production of vesicles (exosomes) of various growth factors, (anti-inflammatory) cytokines, signaling proteins and mitochondria which give rise to recovery of affected neurons and to proliferation of endogenous paraventricular-situated neural stem cells to form oligodendrocytes, astrocytes and neurons.

Increasing experimental evidence shows that regeneration of the injured immature brain with stem cell-based therapies is promising and may serve as an effective treatment strategy. Stem cells have an intrinsic potential for self-renewal and can differentiate into several cellular phenotypes (Fleiss et al., 2014). Given their pluripotent capacity, embryonic stem cells seem the most obvious choice for repair of brain injury, but can induce formation of teratoma after transplantation. Their clinical application raises therefore considerable ethical concerns. This is also true for multipotent neural stem cells: although very attractive given their possibility to derive all neural lineages, their accessibility in humans is limited because they carry also a substantial risk for tumor formation (Comi et al., 2008). Among all progenitor cells, the mesenchymal stem (or stromal) cell (MSC) is at this moment the most optimal choice for near-future use in (preterm) neonates because of the evident neuroregenerative properties and favorable immunological profile and, not for the least, of its favorable safety profile (Uccelli et al., 2008; Fleiss et al., 2014). MSCs are considered to adapt their secretome, after which paracrine signaling results in endogenous brain repair rather than direct cell replacement through MSC differentiation (Qu et al., 2007; van Velthoven et al., 2011). Paracrine effects of MSCs include many growth factors such as insulin-like growth factor (IGF-1), brain-derived neurotrophic factor (BDNF), glial-derived neurotrophic factor (GDNF), and vascular endothelial growth factors (VEGF) (Kizil et al., 2015; Ophelders et al., 2016; Bennet et al., 2018a). These factors can promote endogenous repair through brain cell formation in the sub ventricular zone as well as boost neuronal and glial cell proliferation, maturation and survival on other regions, Moreover, MSCs are shown to secrete anti-inflammatory cytokines, involved in reduction of neuroinflammation (Figure 3). Upregulation of neoneurogenesis and downregulation of genes involved in inflammation after MSC transplantation has been reported in a review (Wagenaar et al., 2017).

MSCs can be administered to the brain *via* several routes: intravenously, intracranially/intrathecally and nasally. The nasal route is non-invasive and seems more effective without loss of MSCs in other organ systems as compared to intravenous administration (Fischer et al., 2009; Wagenaar et al., 2017). In a neonatal stroke model in mice pups substantial beneficial effects on infarction size, motor function and cognition were demonstrated (Wagenaar et al., 2017). The nasally administered MSC cells were no longer detectable 3 days after the implantation, minimizing the risk for Graft-versus Host Disease and tumor growth (Donega et al., 2014). This is confirmed by a long-term safety study of our group (Donega et al., 2015). Moreover human trials on MSC therapy in adults and children did not provide evidence for serious long-term effects (Lalu et al., 2012). An important advantage of MSC-based cell therapy is that autologous as well as allogeneic transplantation can be applied. Autologous intravenous MSC-transplantations, mostly derived and cultured from MSC-rich umbilical cord tissue or cord blood, as well as allogeneic MSCs (see below) are already reported for clinical use in neonatal medicine (Chang et al., 2014; Cotten et al., 2014). A detailed review concerning stem cell-based therapy in neonatology is beyond the scope of this review but is summarized in several recent reviews (Wagenaar et al., 2017; Gronbach et al., 2018; Niimi and Levison, 2018; Vaes et al., in preparation).

### Stem Cell-Therapy and PIVH

Experimental studies reported that cord-derived MSCs substantially attenuated reactive gliosis and cell death which went along with an increase of brain-derived neurotrophic factor (BDNF) (Mukai et al., 2017). Further study showed that MSC-derived BDNF secretion was indeed a critical paracrine factor playing a central role in the attenuation of PIVH-induced brain injury (Ahn et al., 2017). Preclinical data pointed to a repairing effect of MSCs on the sequelae of severe PIVH (Park et al., 2017). Ahn et al showed that in preterm rat pups (P4), in which severe IVH was induced, intraventricularly transplanted human umbilical cord-derived MSCs attenuated posthemorrhagic ventricular dilatation and the area of brain injury (Ahn et al., 2013). They also showed that the window of effective treatment was at least up to 2 days after induction of brain damage (Park et al., 2016).

Clinical experience is still scarce. Some investigators consider DCC as a form of autologous cord blood transplantation since the number of nucleated cord cells in the newborn which also contain pluripotent stem cells increase (Bayer, 2016). A recent small study from Poland in which very preterm infants were given autologous umbilical cord blood showed significantly higher concentrations of growth factors (among them insulin growth factor, epidermal growth factor and stem cell factor), whereas (severe) PIVH incidence seemed lower in the transplanted group as compared to a control group (Kotowski et al., 2017). Although not directly related to the immature brain, a Korean safety and feasibility study in extremely preterm infants to lower the risk of bronchopulmonary dysplasia with allogeneic cord-derived MSCs (endotracheal administration) reported that allogeneic MSC transplantation seemed safe and well-tolerated by the infants (Chang et al., 2014). A safety and efficacy study of the same group is currently including patients with PIVH *(* *ClinicalTrial.gov* *: NCT02673788).*

Although MSC transplantation seems very promising, it may be clear that further clinical research is mandatory to proof its efficacy to attenuate the consequences of (severe) PIVH. In particular, optimization of dosing of MSCs, the preferred type of MSCs (cord-derived vs bone marrow-derived; (Chen et al., 2009)) and most optimal route of administration are important pending questions, which have to be elucidated.

### Stem Cell-Therapy and Diffuse WMI

Treatment with MSCs in preterm neonates with or at risk for dWMI provides us with an exciting and potentially powerful therapy to reduce or even prevent damage to the vulnerable white matter of the preterm neonate. Experimental studies in which perinatal insults as inflammation and hypoxia-ischemia are used separately or in combination showed us already that the paracrine factors secreted by the MSCs promote oligodendrocyte lineage specification, myelination and maturation (Chen et al., 2010; Jadasz et al., 2013; Jellema et al., 2013; Li et al., 2016; Drommelschmidt et al., 2017). It remains to be proven whether MSC-induced endogenous repair mechanisms also lead to substantial positive effects in diffuse WMI of the preterm infant in whom the interplay of inflammation and hypoxia-ischemia appears to be most relevant. Further research is emerging and mandatory.

## Author Contributions

All authors listed have made a substantial, direct and intellectual contribution to the work, and approved it for publication.

### Conflict of Interest Statement

The authors declare that the research was conducted in the absence of any commercial or financial relationships that could be construed as a potential conflict of interest.
